# The impact of revised definitions on the epidemiology of status epilepticus: A population‐based study

**DOI:** 10.1111/epi.18535

**Published:** 2025-07-21

**Authors:** Meike Menche, Clara Jünemann, Jens‐Peter Reese, Martin Jünemann, Michael Teepker, Christoph Best, Christian Roth, Ingrid Sünkeler, Elisabeth Pryss, Felix Rosenow, Adam Strzelczyk, Leona Möller, Lena Habermehl, Panagiota‐Eleni Tsalouchidou, Ole Simon, Andre Kemmling, Christopher Nimsky, Lars Timmermann, Katja Menzler, Susanne Knake

**Affiliations:** ^1^ Epilepsy Center Hesse, Department for Neurology, University Hospital Marburg Philipps University Marburg Germany; ^2^ Faculty of Health Sciences TH Mittelhessen University of Applied Sciences Giessen Germany; ^3^ Department for Neurology, University Hospital Giessen Justus‐Liebig‐University Gießen Germany; ^4^ Hardtwald Clinic for Neurology Bad Zwesten Germany; ^5^ Vitos Clinic for Neurology Weilmünster Germany; ^6^ Department of Neurology Klinikum Kassel Kassel Germany; ^7^ BDH Clinic for Neurology Braunfels Germany; ^8^ Department of Internal Medicine Kreiskrankenhaus Frankenberg Frankenberg Germany; ^9^ Epilepsy Center Frankfurt Rhine‐Main, Center of Neurology and Neurosurgery University Medical Center Frankfurt, Goethe‐University Frankfurt Frankfurt am Main Germany; ^10^ Department of Epilepsy, Cleveland Medical Center University Hospitals Cleveland Ohio USA; ^11^ Department of Neuroradiology, University Hospital Marburg Philipps University Marburg Germany; ^12^ Department of Neurosurgery, University Hospital Marburg Philipps University Marburg Germany; ^13^ LOEWE‐Research‐Cluster for Advanced Medical Physics in Imaging and Therapy (ADMIT) TH Mittelhessen University of Applied Sciences Giessen Germany

**Keywords:** case–fatality rate, epidemiology, epilepsy, etiology, incidence, mortality, status epilepticus

## Abstract

**Objective:**

Status epilepticus (SE) represents one of the most common neurological emergencies. The International League Against Epilepsy (ILAE) redefined SE duration thresholds from 30 to 5 min for convulsive SE in 2015. We conducted a prospective population‐based study to determine SE incidence and outcomes under the revised criteria and compared findings with historical data using the 30‐min definition.

**Methods:**

A prospective, population‐based cohort study was conducted over a period of 18 months to determine the incidence of SE in Germany, replicating the methodology of a first study conducted in this region in 1999. The study included all adults residing within the 35‐postcode area, with participation from all regional hospitals and emergency departments. SE cases were prospectively identified and reported. To ensure comparability with the historical data, the analysis focused on the Primary Service Area (PS‐Area)—the direct catchment region of the University Hospital Marburg.

**Results:**

A total of 180 adults with SE (96 men, mean age 66.47 years, SD ± 18.5 years, range: 20–94 years). The crude annual incidence in the PS‐Area increased from 15.8/100 000 (95% confidence interval [CI] 11.2–21.6) in 1999 to 29.4/100 000 adults (95% CI 20.5–40.0). It was higher in men than in women (30.9 vs 28.1/100 000, *p* = .11) and in patients ≥60 years (68.5 vs 13.5/100 000; *p* < .0001). The calculated age‐ and gender‐adjusted incidence was 32.5/100 000 in the PS‐Area. The case–fatality rate was 5.77% (95% CI 1.2%–12.7%) and the crude annual cause‐specific mortality rate per 100 000 is 1.70 (95% CI 0.21–4.73). In 53% SE was the first seizure episode; only 47% had a history of epilepsy. When extrapolating these findings to the entire German population, there were at least 20 000 cases of SE with 1000 associated deaths annually.

**Significance:**

These findings provide new epidemiologic evidence that the incidence of SE has increased by ~12% with the adoption of the ILAE 2015 definition, underscoring the impact of updated diagnostic criteria on epidemiological estimates.

**Key points**

- Changing the operational definition of status epilepticus (SE) from 30 min to 5 min resulted in a significant increase in the reported crude annual incidence (from 15.8/100 000 to 29.4/100 000)
- The 30‐day crude case–fatality rate (CFR) was 5.77% (95% confidence interval [CI] 1.2%–12.7%); the crude annual cause‐specific mortality rate was 1.7/100 000.
- The data provide the first prospective epidemiological data on SE using the shorter operational definition of 5 min in Germany

## 1 INTRODUCTION

Status epilepticus (SE) is defined as “a condition resulting either from the failure of the mechanisms responsible for seizure termination or from the initiation of mechanisms leading to abnormally prolonged seizures.”^1^ SE is one of the most severe neurological emergencies and manifestation of epilepsy, associated with a high case–fatality rate (CFR) and high socioeconomic costs.^2^, ^3^, ^4^, ^5^ SE may have long‐term consequences, including neuronal death or injury and alteration of neuronal networks, depending on the type and duration of seizures. In 2015 the International League against Epilepsy (ILAE) published a new classification of SE.^6^, ^7^ The definition of SE for generalized convulsive status epilepticus (CSE) was modified from 30 min to 5 min, and 10 min for focal SE and absence SE.^1^, ^8^ The German guidelines were revised to define each seizure of prolonged seizure activity exceeding 5 min as SE, mandating urgent treatment initiation.^9^, ^10^

The incidence of SE has been determined primarily by population‐based studies based on the 30‐min definition, suggesting an incidence per year of 15.8/100 000 (95% confidence interval [CI] 11.2–21.6) in Germany,^11^ 9.9 to 10.3/100 000 in Switzerland,^12^ 27.2/100 000 in Italy,^13^, ^14^, ^15^ 3.5 to 41/100 000 in North America,^16^, ^17^, ^18^, ^19^, ^20^ 1.3 to 5.2/100000 in Asia,^21^, ^22^ and 10.8/100 000 in Africa.^23^ Leitinger et al. conducted a population‐based retrospective study using the new ILAE definition in Austria, with an incidence rate of 36.1 per 100 000 per year.^24^ One study in New Zealand conducted a prospective population based study across all age groups using a definition of SE of seizures lasting 10 min or longer finding an age‐adjusted incidence of 25.54/100 000.^4^

Since the temporal definition was modified from a 30‐min to a 5‐min definition, no prospective population‐based study has been conducted to evaluate the impact of this change on the incidence and mortality of SE in Germany.

## 2 MATERIALS AND METHODS

Data for the present study were collected within the same geographical region (identical postcode area) and using the same study design as the 1999 investigation on the incidence of SE in adults in Germany.^11^ To facilitate direct comparison of incidence rates, the study design and analytical methods were replicated. This allowed evaluation based on both the former 30‐min definition and the current 5‐min definition of SE.

Seven hospitals within the postcode area 35 and seven hospitals in the surrounding regions participated in the study. All 14 hospitals were contacted daily and asked to report any case of SE to minimize the risk of underreporting. Of these, seven hospitals reported patients meeting the inclusion criteria between September 2018 and February 2020. Not all of the participating hospitals had an emergency department, and several smaller institutions lacked a neurology consultant. Consequently, it is not unexpected that seven hospitals did not report any eligible cases. All identified patients were enrolled in the study within 3 days of notice. Data collection was conducted using a standardized questionnaire previously employed in the 1999 study.^11^ A follow‐up was conducted via telephone or email 30 days after the patient's discharge. The hospital database of the University Hospital Marburg were checked retrospectively for all discharge diagnoses “Status epilepticus” using the German Modification of the International Classification of Diseases, 10th Revision (ICD‐10‐GM), code G41.

The number of patients identified was compared to the number of patients enrolled in order to prevent underreporting.

To facilitate direct comparison with the 1999 data, the incidence within the Primary Service Area (PS‐Area) was calculated using the same methodology as in the historical study. In this study, data from the direct catchment area of the University Hospital Marburg were considered more accurate and were associated with a lower rate of underreporting in the previous analysis.^11^

The most recent ILAE classification of SE and the Operational Classification of Seizure Types was used to classify the seizure and SE types.^1^, ^8^, ^25^ In accordance with the German guideline, patients were included on the basis of their clinical presentation or on electroencephalography (EEG) data, with an SE lasting longer than 5 min, regardless of the status form.^9^

A total of 180 patients were included, of whom 102 had generalized CSE. The second largest group was focal SE, with 21 single‐focal and 22 complex‐focal cases. Non‐convulsive SE was recorded in 19 cases, with only three patients showing absence status.

Survivors were defined as patients who were alive 30 days after the onset of SE.

**FIGURE 1:**
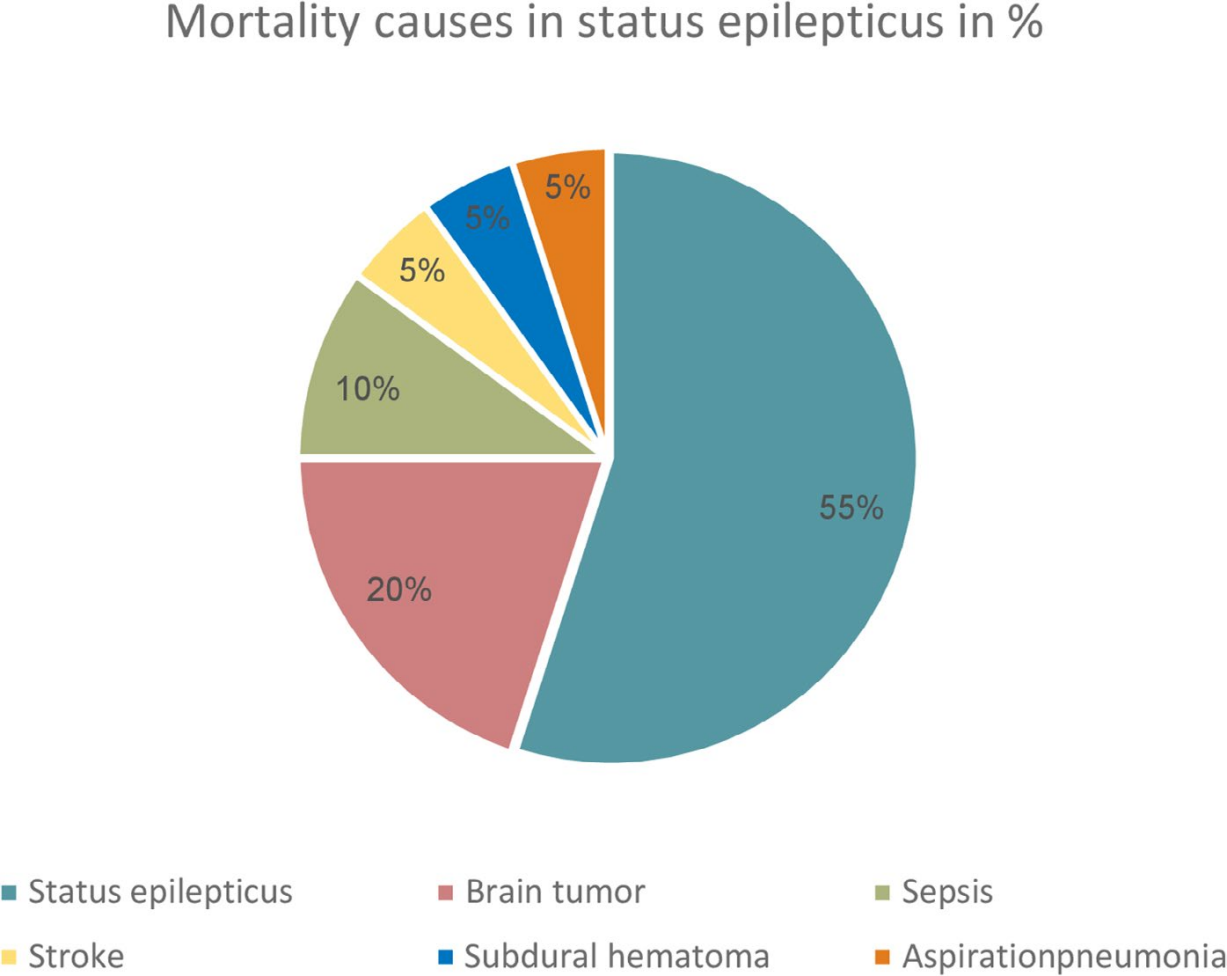
Mortality causes in status epilepticus.

20 of the 180 patients died, more than half of the deaths were related directly to SE (*n* = 11). Four of the 20 patients died of tumor‐related disease, the other deaths were due to sepsis, aspiration pneumonia, stroke, or subdural hematoma (Figure 1).

### 2.1 Etiology

Etiology was classified into six groups according to the ILAE classification: structural (e.g., stroke, tumor, and previous brain surgeries), genetic, infectious, metabolic, immune, and unknown.^8^ These categories included acute etiologies occurring within 7 days of the potential brain lesion and remote etiologies causing seizures later than 7 days after the potential etiology. If there was no identifiable etiology, SE was classified as unknown.

The epidemiological data were obtained from the Federal Statistical Office in Berlin and referred to the year 2019.^26^ In 2019 the population of the area under investigation included 981 068 adults. The data indicated a ratio of 51% women to 49% men. The demographic profile of the postcode area under examination exhibited a slight tendency toward younger age groups than the national average, with a population comprising 329 430 individuals who were 60 years or older. A comparison of the population in the area with that in 1998 revealed a population increase of 237 783 inhabitants, representing a population growth of 23%. This was accompanied by a notable increase in the proportion of the population 60 years and older, from 27% in the 1999 study to 34% in the actual data. The sex distribution remained relatively constant, with 51% female.

A total of 180 patients were included in the study; all met the inclusion criteria and were at least 18 years of age, resided in the specified 35 postcode area, and had experienced a seizure lasting at least 5 min.

The patients were reported to the study team and recorded within 3 days using a standardized questionnaire developed for the prospective study on the incidence of SE from 1999.^11^, ^27^ Items such as demographic data, date and length of the SE, past medical history, etiology of the SE, results of diagnostic procedures, used therapeutic strategies including pre‐hospital medication and antiseizure medication (ASM) therapy data on the current hospital stay. Outcome at discharge as well as 30 days post‐discharge were assessed using the modified Rankin scale (mRS).^28^

Prior to enrollment, all patients or their legal guardians provided written informed consent. The study was performed in accordance with the Strengthening the Reporting of Observational Studies in Epidemiology (STROBE) guidelines for observational studies in epidemiology and received ethical approval from the Ethics Committee of Philipps University of Marburg (reference number: Studie 42/18).^29^

Ten patients were excluded from the study, as they did not meet the inclusion criteria or had an incomplete medical history.

### 2.2 Statistical analysis

The number of adults registered in the post code area 35 was provided by the Federal Statistical Office.^26^ The crude annual incidence and CIs were calculated by dividing the number of all first time episodes of SE by the number of the population at risk (here: all adults ≥18 years residing in this area) using a single rate approach. The Clopper–Pearson CI was used to calculate the 1‐year incidence rate in that area. The age‐ and gender‐adjusted incidence was calculated to interpolate more representative results for Germany using the same formula and statistical analysis used in the previous study for this region.^27^

For comparison with the 1999 incidence study, the incidence within the PS‐Area was calculated. Group comparisons were performed using non‐parametric statistical methods, specifically the chi‐square test.

## 3 RESULTS

A total of 180 adult patients were included in the study. Patient characteristics are summarized in Table 1. In all cases, the duration of SE exceeded 5 min. The total duration of the SE episodes was documented in 111 cases (61.7%), with a mean duration of 509.84 min. Among these, 53 patients experienced refractory or super‐refractory SE requiring intubation. However, no reliable data on the duration of SE were available in 38.3% of patients (Table 1).

**TABLE 1:** Clinical characteristics and demographic data of all included patients with status epilepticus (SE).

Patient characteristics	
Number of patients	180
Age	Range: 20–94 years
Age groups	
<60 years	52 (28.89%)
≥60 years	125 (69.44%)
Unknown	3 (1.67%)
Mean age (SD), years	66.47 (18.5)
Median age (IQR), years	70 (22)
Sex distribution	
Male patients	96 (53.33%)
<60 years	33 (34.38%)
Female patients	81 (45%)
<60 years	19 (23.46%)
Non‐refractory SE	55 (30.56%)
Refractory SE/super‐refractory SE	56 (31.11%)
Mean SE duration	509.84 min (range: 5–7200 min)

**FIGURE 2:**
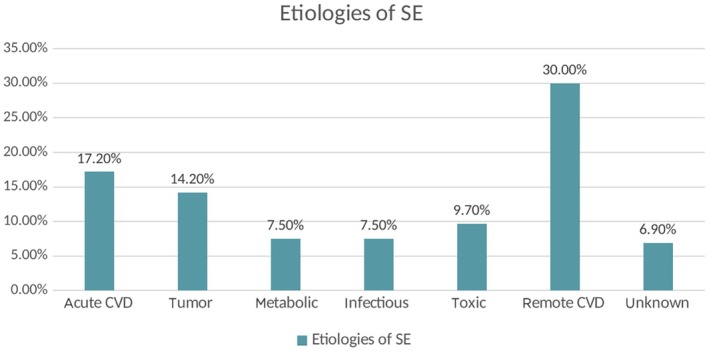
Etiologies of status epilepticus.

More than half of the patients presented with SE as the first seizure ever; only 47% of the patients had a history of epilepsy, and 15.2% of these patients had a history of epilepsy but had been seizure‐free for over 1 year. Among all patients, 17.7% had a history of SE. A total of 73.5% of patients required intensive care therapy, and 29% of the study population required intubation. In the initial 30‐day period following the onset of SE, 20 out of the 180 patients died, resulting in a crude 30‐day CFR of 11.11% (95% CI 6.2%–14.5%). To ensure comparability between the CFR and incidence of the present study and the 1999 study, the CFR and incidence were calculated for the PS‐Area, which was the basis for the results reported in the previous study. It was assumed that there was no underreporting in this area. In the PS‐Area, the crude 30‐day CFR was notably lower: 5.77% (95% CI 1.2%–12.7%). Acute or remote cerebrovascular disease (CVD) were the most frequent etiologies, followed by brain tumors or metastases. An acute etiology was identified in 56.1% of cases, mainly due to acute CVD (17.2%); tumors (14.2%); or metabolic, infectious, and toxic disorders (7.5%, 7.5%, and 9.7%). Remote etiologies accounted for 37% of cases, predominantly remote CVD older than 4 weeks (30%) (Figure 2).

The mean age of patients with remote etiology was 73.15 years, which was higher than in the acute symptomatic SE group (69.72 years). The five most common comorbidities were cerebrovascular disease (42.6%), diabetes mellitus (17.4%), dementia (15.5%), tumor disease (14.8%), and renal insufficiency (13.6%).

Refractory SE (RSE) and super‐refractory SE (SRSE) were reported in 31.11%. At discharge, the SRSE group had a higher level of disability (mRS > 3) in 70% of cases, significantly more than the RSE (41%) and non‐refractory SE (NRSE) (37%) groups.

The crude annual incidence in the PS‐Area was 29.4 per 100 000 adults (95% CI 20.5–40); it was higher in men than in women (30.9 vs 28.1) and in patients 60 years of age or older (68.5 vs 13.5/100 000; *p* < .0001).

The age‐ and gender‐adjusted SE incidence rate was 32.5 per 100 000. The incidence rate was significantly higher in the population 60 years of age or older (*p* < .0001) and higher in men (*p*‐value <.05). The crude case fatality rate after 30 days of SE was 5.77% (95% CI 1.2%–12.7%) in the PS‐Area.

**FIGURE 3:**
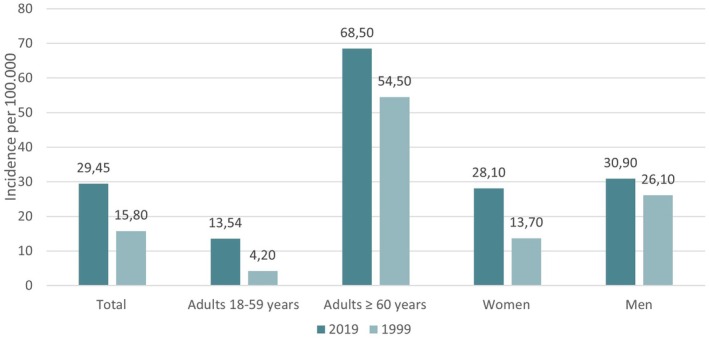
Crude annual incidence of status epilepticus (SE) compared with the crude incidence of SE in the same area using the previous 30‐min definition.

Extrapolating these findings to the entire country, at least 20 000 SE cases with more than 1000 associated deaths annually may be expected in Germany (Figure 3).

## 4 DISCUSSION

The crude annual incidence of SE in the PS‐Area of Marburg was 29.4/100 000 adults per year (95% CI 20.5–40.0); the age‐ and gender‐adjusted SE incidence rate was 32.5 per 100 000. Following the revision of the operational definition of SE from a 30 min to a 5 min threshold, this prospective, population‐based study identified a significant increase in the crude SE incidence rate to 29.4/100 000 (95% CI 20.5–40.0), compared to 15.8 per 100 000 (95% CI 11.2–21.6) reported in 1999.

A study by Leitinger et al. was the first to use the ILAE 2015 criteria in a population‐based setting, providing updated and higher incidence rates for SE: They reported an overall age‐ and sex‐adjusted incidence of first episodes of SE of 36.0 per 100 000 per year, similar to the one reported here of 32.5 per 100 000 per year. Leitinger et al. found that the adoption of the ILAE 2015 definition increased the observed incidence of SE by about 10% in the Salzburg area compared to the previous definitions. However, it is noteworthy that the recorded incidences did not differ significantly between the two studies. A notable finding observed in the current study was the lower mortality rate. This finding may result from the PS‐Area around the University Hospital of Marburg, which is regionally small compared to larger regions or cities and has immediate access to a neuro‐ICU (intensive care unit). Furthermore, our study population was slightly younger, with a mean age of 66.47 years.^24^

The 30‐day CFR in the PS‐Area was 5.77%. For comparison with the historical data, the CFR for the entire postcode area 35 was calculated, demonstrating a 30‐day CFR of 11.11% (95% CI 6.2%–14.5%), compared to 9.3% reported in 1999. These findings suggest a temporal decline in CFRs in the PS‐Area.^30^, ^31^, ^32^ The incidence in the population 60 years of age or older increased significantly between the two studies (68.5 vs 54.5 per 100 000), and the increase in the population younger than 60 years is also notable (13.5 vs 4.2 per 100 000). When these results are extrapolated to the entire country, at least 20 000 SE cases with 1000 associated deaths can be expected annually in Germany.

Prior research has demonstrated a higher prevalence of SE among older age groups as well as a higher incidence in men compared to women.^11^, ^12^, ^15^, ^33^, ^34^, ^35^, ^36^, ^37^, ^38^ In comparison to the 1999 study, we observed an increased incidence among women. This finding may reflect improved health care access and advances in diagnostic capabilities for female patients. Nevertheless, further research is warranted to elucidate the role of gender in the incidence and etiology of SE.^27^, ^39^

As in most epidemiological studies on SE, underreporting of focal and nonconvulsive SE, potentially due to underdiagnosis, remains a concern.^40^, ^41^, ^42^ However, such sources of underreporting cannot fully be excluded and may have influences in both the 1999 study and in the current one. On the other hand, factors such as the inclusion of recurrent episodes and the diagnostic time criterion of 5 min might have led to overestimation of SE for all types of SE.^43^

To enable a direct comparison with the 1999 study, the study design was deliberately maintained; only the 5‐min diagnostic criterion differed, following the new national guidelines.^11^

The observed increase in incidence and the decrease in the 30‐day CFR in the PS‐Area are not unexpected, as we included patients who were likely missed in the previous study. The mean duration of NRSE in this study was 32 min, suggesting that a significant number of patients would not have been diagnosed or treated according to the previously applied 30‐min diagnostic threshold for SE. As a consequence of the earlier diagnosis, patients will be receiving treatment at an earlier stage. It has been demonstrated in previous studies that prompt and efficacious treatment has a substantial impact on prognosis and quality of life.^44^, ^45^, ^46^, ^47^, ^48^, ^49^, ^50^, ^51^

The prevailing etiology identified in both the present study and the 1999 study was cerebrovascular disease. In 1999 acute ischemia was responsible for 14% of cases, whereas remote ischemia accounted for 36%, which is comparable to the respective 17% and 30% observed in the actual study (Figure 2).^52^

Notably, only 33% of the enrolled study patients received adequate therapy within the first 30 min. The implementation of national treatment guidelines, coupled with improved access to well‐tolerated intravenous antiseizure medications, should further enhance early intervention and, consequently, prognosis.^53^, ^54^, ^55^, ^56^

The duration of SE is known as a predictor of long‐term mortality, which supports the clinical usefulness of the new, shorter operational definition.^57^ Our findings are supported by other data from the UK, where the number of patients admitted to the ICU with SE tripled from early 2000 to early 2010, whereas acute hospital mortality decreased from 8.1% to 4.4% over the same period.^58^

However, this study has some limitations. The use of a historical control group may introduce some bias. A comparison over a long period of time (here 20 years) may not take into account changes in health care systems, diagnostic capabilities, and treatment approaches. The present study observed a population growth of 23% in the postcode area over the last two decades. Despite comparable basic structures such as age and sex distribution, concerns of whether the study area is identical to that of the previous study may be considered. Furthermore, the increased use of and access to emergency EEG improved the detection of NCSE, which may have been underreported in the past. In addition, variations in data quality and the use of different treatment algorithms and emergency medication favoring a more aggressive initial treatment may have affected the identification and outcome. Other factors, such as changes in demographics, general improvements in emergency care, and facilitated access to specialized treatment might also influence incidence and mortality. We used a prospective, population‐based design and tried to replicate the exact study design of the previous study in exactly the same area 20 years earlier.

More research is needed to understand the impact of these factors on epidemiology and outcomes. Future studies should also investigate the long‐term effects of early intervention and assess the effectiveness of different treatment options in different patient populations.

## CONFLICT OF INTEREST STATEMENT

Meike Menche: none. Clara Jünemann: received traveling grants from the ILAE, DGfE (Deutsche Gesellschaft für Epileptologie), and DGN (Deutsche Gesellschaft für Neurologie). Jens‐Peter Reese: none. Martin Jünemann: none. Michael Teepker: none. Christoph Best: none. Christian Roth: none. Ingrid Sünkeler: none. Elisabeth Pryss: none. Felix Rosenow: reports personal fees and travel expenses from Angelini Pharma, Desiitn Pharma, Eisai GmbH, Stoke Pharmaceuticals, Jazz Pharma, Takeda, and UCB, and research support from German Research Foundation, The LOEWE Programme of the state of Hesse, the Detlev‐Wrobel Fonds for Epilepsy Research Frankfurt, the Reiss Stiftung, the Dr. Senckenbergische Stiftung, the Kassel Stiftung, the Chaja Stiftung, and the Ernst Max von Grunelius. Adam Strzelczyk: reports personal fees and grants from Angelini Pharma, Biocodex, Desitin Arzneimittel, Eisai, Jazz (GW) Pharmaceuticals, Takeda, UCB (Zogenix) Pharma, and UNEEG Medical. Leona Möller: Marburg University Research Academy (MARA) und Stiftung P.E. Kempkes. Lena Habermehl: received traveling grants from GW Pharma, Zogenix, and Angelini Pharma. Panagiota‐Eleni Tsalouchidou received a the Otfrid‐Foerster‐Grant from the DGfE. Ole Simon: none. Andre Kemmling, Christopher Nimsky, and Lars Timmermann: received occasional payments as a consultant for Boston Scientific; L.T. received honoraria as a speaker on symposia sponsored by Boston Scientific, AbbVIE, Novartis, Neuraxpharm, Teva, the Movement Disorders Society, und DIAPLAN. The institution of L.T., not L.T. personally, received funding by Boston Scientific, the German Research Foundation, the German Ministry of Education and Research, the Otto‐Loewi‐Foundation, and the Deutsche Parkinson Vereinigung. Neither L.T. nor any member of his family holds stocks, stock options, patents or financial interests in any of the above mentioned companies or their competitors. L.T. served as the president of the German Neurological Society without any payment or any income. Katja Menzler speaker's honoraria and consultant fees from Bial, Jazz Pharma, UCB, and Eisai. Susanne Knake received speaker's honoraria from Bial, Eisai, Desitin, Kanso, Merck Serono, and UCB Pharma. We confirm that we have read the Journal's position on issues involved in ethical publication and affirm that this report is consistent with those guidelines.

## Data Availability

Research data are not shared.
